# Microarray of surface-exposed proteins of *rickettsia heilongjiangensis* for serodiagnosis of Far-eastern spotted fever

**DOI:** 10.1186/1471-2334-14-332

**Published:** 2014-06-17

**Authors:** Yong Qi, Wenping Gong, Xiaolu Xiong, Jiafu Jiang, Yawei Wang, Jun Jiao, Changsong Duan, Bohai Wen

**Affiliations:** 1 State Key Laboratory of Pathogen and Biosecurity, Beijing Institute of Microbiology and Epidemiology, 20 Dong-Dia-Jie Street, Fengtai, Beijing, China

**Keywords:** Far-eastern spotted fever, *Rickettsia heilongjiangensis*, Protein microarray, Serological diagnosis

## Abstract

**Background:**

Far-eastern spotted fever (FESF) is an important emerging infectious disease in Northeast Asia. The laboratory diagnosis of FESF in hospitals is mainly based on serological methods. However, these methods need to cultivate rickettsial cells as diagnostic antigens, which is both burdensome and dangerous.

**Methods:**

Eleven surface-exposed proteins (SEPs) were identified in our previous study and their recombinant proteins (rSEPs) fabricated on a microarray were serologically analyzed with seventeen paired sera from patients suffered from FESF in this study.

**Results:**

All the rSEPs showed sensitivities of between 53% and 82% to acute-phase sera and of between 65% and 82% to convalescent-phase sera, and all the rSEPs except rRplA showed specificities of between 80% and 95%. The combination assay of two, three, or four of the four rSEPs (rOmpA-2, rOmpB-3, rRpsB, and rSdhB) showed better sensitivities of between 76% and 94% to the acute-phase sera or between 82% and 100% to the convalescent-phase sera and acceptable specificities of between 75% and 90%.

**Conclusions:**

Our results suggest that the four rSEPs are more likely candidate antigens for serological diagnosis of FESF.

## Background

*Rickettsia heilongjiangensis*, a spotted fever group (SFG) rickettsia isolated from ticks in 1983 in Heilongjiang Province of China [1], is the causative agent of Far-eastern spotted fever (FESF). FESF has been considered as an important emerging infectious disease in Northeast Asia for this rickettsiosis has been diagnosed in Northeast of China [2], east-Siberian and far-eastern regions of Russia [3,4], and Japan [5]. Most of the patients naturally infected by *R. heilongjiangensis* had fever, chills, headache, dizziness, myalgias, arthralgias, and anorexia after an incubation period of 4 to 7 days, and later most of the patients appeared with a macular or maculopapular rash at the site of tick attachment and lymphadenopathy regional to the eschar [4]. Almost half of the patients had hepatomegaly accompanied with an increased alanine aminotransferase and/or aspartate aminotransferase activity [4]. In a murine model, *R. heilongjiangensis* caused severe systemic infection with lesions in multiple organs (liver, lung, and brain) [6].

So far, the laboratory diagnosis of rickettsioses in hospitals is mainly based on serological methods although cell culture and molecular tools like PCR or real-time PCR are applied [7]. Immunofluorescence assay (IFA) is the gold standard and is used as a reference technique in most laboratories [7]. A gold diagnostic standard for rickettsioses, IFA followed by real-time PCR, has been built in the French National Reference Center (FNRC) [8]. However, IFA needs to cultivate rickettsial cells as diagnostic antigens, which is both burdensome and dangerous.

Some efforts have been focused on screening rickettsial proteins as serodiagnostic antigens of rickettsioses [9,10]. Kowalczewska et al. characterized 20 rickettsial recombinant proteins by enzyme-linked immune sorbent assay (ELISA) with sera from patients infected by *R. typhi* or *R. conorii*[10], in which, many surface-exposed proteins (SEPs) like Adr2, Omp1, PLD, RickA, Sca1, Sca10, and Sca13 were used. In fact, many SEPs have been found to be suitable as diagnostic antigens, such as a 56 kDa outer membrane protein in detection of *Orientia tsutsugamushi* infection [11,12] and a surface protein Pap31 in detection of *Bartonella bacilliformis* infection [13]. These findings indicate that SEPs are more likely to be diagnostic candidates. In our previous study, 24 SEPs of *R. heilongjiangensis* were identified and their recombinant proteins (rSEPs) fabricated on a microarray were serologically analyzed and eleven of them were recognized as major seroreactive proteins and potential candidate antigens for serological diagnosis of FESF by sera from mice experimentally infected with *R. heilongjiangensis*[9]. Also, these rSEPs in microarray assay showed a higher specificity in recognizing *R. heilongjiangensis*-infected mouse sera compared with that in ELISA [9]. In the present study, these rSEPs fabricated on a protein microarray were assayed with paired sera from FESF patients during the acute and convalescent phase.

## Methods

### Patient sera

FESF was diagnosed in patients by PCR using whole blood [14] as well as clinical symptoms consistent with tick-bite fever, multiple inoculation eschars and cutaneous rash in hospital. IgG antibody titers of patient sera were determined by IFA with *R. heilongjiangensis* antigen as described previously [9]. Each case of FESF was confirmed by a single serum with the specific IgG titer of ≥1:128 or the paired sera with a fourfold or greater increase of the specific IgG titers. The paired sera were collected from 17 patients suffered from FESF during the acute and convalescent phase. The acute-phase sera were collected from the patients at the date of onset of illness, and the convalescent-phase sera were collected from the same patients approximately two weeks after the first sampling. Also, 20 sera, collected from acute febrile patients with uncertain diagnoses and their titers of IgG antibodies to *R. heilongjiangensis* being determined to be less than or equal to 1:8 in IFA, were used as negative control or as reference sera to assess diagnostic specificity of the microarray assay in this study.

All of the patient sera were obtained from a hospital in northeast China. The serum samples of patients were collected as part of the routine management of patients without any additional sampling. All patients gave their informed consent and all patient data were deidentified. The Institutional Review Board of the Beijing Institute of Microbiology and Epidemiology approved the research involving human materials.

### Preparation of recombinant proteins

Eleven rSEPs of *R. heilongjiangensis*, including rGroEL, rOmpA-2, rOmpB-3, rPrsA, rRplA, rRplY, rRpsB, rSdhB, rSurA, rYbgF, and rRh054_02285, were used in the present study. The preparation and purification of these recombinant proteins were described in our previous study [9,15].

### Immunoblotting assay

The purified rSEPs were immunoblotted with the paired sera from one patient with FESF. Briefly, rSEPs separated by SDS-PAGE were transferred to polyvinylidene difluoride (PVDF) membrane. The PVDF membrane was blocked with 1% [w/v] bovine serum albumin (BSA) in phosphate buffer saline (PBS, containing 8.1 mM Na_2_HPO_4_, 1.9 mM NaH_2_PO_4_, and 154 mM NaCl) at pH 7.4 overnight. Then, rSEPs on the PVDF membrane were incubated with the acute- or convalescent-phase serum (1:250 dilution) that was previously neutralized with *E. coli* lysate (5 mg/ml) for 1 h. After three washes in PBST (pH 7.4 PBS containing 0.05% [v/v] Tween 20), the PVDF membrane was incubated with horseradish peroxidase (HRP)-conjugated goat anti-human IgG (1:5 000 dilution; Beijing CoWin Biotech, Beijing, China) for 1 h. Following an additional three washes in PBST, the PVDF membrane was developed using a diaminobenzidine (DAB) kit (Boster, Wuhan, China).

### Fabrication of protein microarray

Each of the purified rSEPs diluted in PBS to a concentration of 0.3 mg/ml was printed on epoxy slides (CapitalBio, Beijing, China) in 5 replicate spots as described previously [16]. Human IgG with serial dilutions (2.5, 5, 10 and 20 μg/ml) was used to fit the internal calibration curves or as positive controls. BSA in PBS or lysate of *E. coli* cells transformed with pET-32a plasmids at a concentration of 0.3 mg/ml was used as negative controls [16]. For quality control, the microarray slides were incubated with mouse anti-His tag IgG-Cy5 (SBA, Birmingham, AL) and the fluorescence intensity (FI) of each protein on the slides was scanned by GenePix Personal 4100A (Molecular Devices, Sunnyvale, CA) and analyzed by GenePix Pro 6.0 software (Molecular Devices, Sunnyvale, CA) [16]. Proteins with a signal-to-background ratio over 3.0 were used for further analysis [16].

### Analysis of proteins on microarrays by patient sera

The rSEPs on the microarray slide were probed using patient sera according to previous descriptions [9]. Briefly, the microarray slide was blocked with 1% [w/v] BSA in PBS overnight. Then each well on the slide was incubated with 50 μl of each patient serum (1:50 dilution) that were previously neutralized with *E. coli* lysate (5 mg/ml) for 1 h [16]. After six washes in PBST, the microarray slide was developed by incubation with goat-anti human IgG-Cy5 (SBA) (1:500 dilution) for 1 h. Following an additional five washes in PBST and a final wash in deionized water, the air dried microarray slide was scanned with a GenePix Personal 4100A scanner and the scanned images were analyzed by GenePix Pro 6.0. The FI value of each protein was calculated by averaging the FI values of five replicate spots, which had been background-subtracted [9].

### Microarray data analysis

Human IgG dose-FI value curves were fitted with linear regression analysis using GraphPad Prism 5 software (GraphPad Software, Inc., San Diego, CA). The relative amounts of specific IgG (RASIgG) to individual rSEPs in each serum were determined by interpolating the calculated FI value with the IgG internal calibration curve [17].

The cutoff value for individual rSEPs in the microarray assay was generated as described previously using Youden’s index [13]. The reaction was considered positive if the RASIgG to one rSEP in any of the patient sera was higher than the cutoff value.

### Statistical analysis

The log-transformed IgG titers to *R. heilongjiangensis* in IFA and the numbers of rSEPs recognized by FESF patient sera in microarray assay were analyzed for potential correlations by linear regression using GraphPad Prism 5 software.

The IgG titer of the convalescent-phase serum divided by the IgG titer of the acute-phase serum was calculated as the increased titer for each paired sera. The increase in RASIgG to each rSEP in each paired sera was calculated as follows: The increased RASIgG = RASIgG to one rSEP in the convalescent-phase serum/RASIgG to the same rSEP in the acute-phase serum.

In paired sera, the correlation between the increase in IgG titers (log transformed) to *R. heilongjiangensis* and the increase in RASIgG to individual rSEPs was analyzed by linear regression using GraphPad Prism 5 software.

## Results

### IgG titers of sera determined by IFA

The IgG titers of 17 paired sera from FESF patients were determined by IFA with *R. heilongjiangensis* antigen. Fourteen of these paired sera showed a fourfold or greater rise in specific IgG titers and the other 3 paired sera showed high specific IgG titers greater than or equal to 1:128 (Table 1).

**Table 1:** The IgG titers of paired sera from patient suffered from FESF in IFA

**Patients no.**	**Acute-phase serum sample**	**Convalescent-phase serum sample**	**Diagnostic criteria**
1	128	512	Fourfold increase
2	64	1024	Fourfold increase
3	256	512	IgG titer ≥128
4	64	256	Fourfold increase
5	32	512	Fourfold increase
6	128	1024	Fourfold increase
7	128	1024	Fourfold increase
8	256	512	IgG titer ≥128
9	32	512	Fourfold increase
10	256	256	IgG titer ≥128
11	64	1024	Fourfold increase
12	64	1024	Fourfold increase
13	64	512	Fourfold increase
14	128	1024	Fourfold increase
15	128	1024	Fourfold increase
16	64	512	Fourfold increase
17	64	1024	Fourfold increase

### Immunoblotting assay

**Figure 1:**
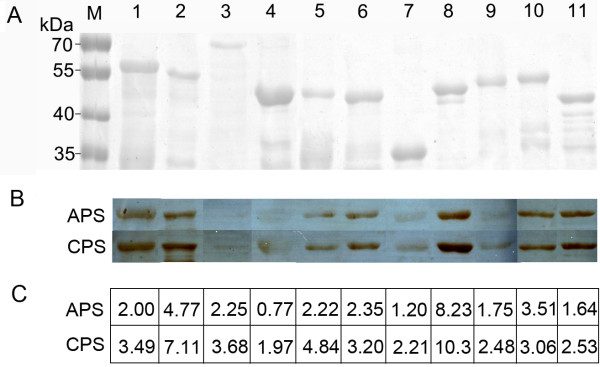
**SDS-PAGE and immunoblotting analysis of the purified rSEPs.** Eleven rSEPs were analyzed with SDS-PAGE **(A)** and immunoblotted with acute-phase sera (APS) or convalescent-phase sera (CPS) of patient 8 **(B)**. The relative amount of specific antibodies to individual rSEPs in APS or CPS of patient 8 was calculated in microarray assay **(C)**. Lanes 1 to 11 refer to rGroEL, rOmpA-2, rOmpB-3, rPrsA, rRh054_02285, rRplA, rRplY, rRpsB, rSdhB, rSurA, and rYbgF, respectively. Lane M refers to protein markers and their relative molecular masses are indicated in kDa on the left.

Eleven rSEPs of *R. heilongjiangensis* were immunoblotted by the acute- or convalescent-phase serum from one of FESF patients (patient 8 in Table 1). As shown in Figure 1, seven rSEPs (rGroEL, rOmpA-2, rRh054_02285, rRplA, rRpsB, rSurA, and rYbgF) were recognized strongly by both acute- and convalescent-phase serum and the rest were recognized weakly. Seven rSEPs (rGroEL, rOmpA-2, rPrsA, rRplA, rRpsB, rSdhB, and rYbgF) showed a stronger staining reaction with the convalescent-phase serum than with the acute-phase serum.

### Quality control of protein microarray

For quality control, the microarray slides printed with rSEPs were incubated with mouse anti-His tag IgG-Cy5 (SBA, Birmingham, AL) and scanned for their FI values. The coefficient of variations (CV) was calculated as the SD of the FI value for each SEP divided by the average FI value. As a result, the within-slide CV (n = 6) and between-slide CV (n = 6) of individual rSEPs on the microarray ranged from 8% to 18%.

### Sensitivity and specificity of rSEPs in microarray assay

**Figure 2:**
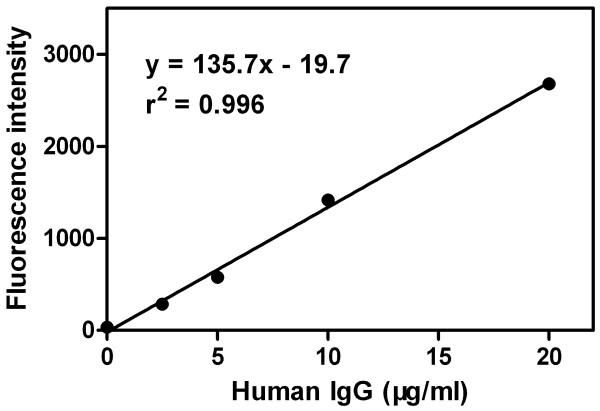
One example of the internal calibration curves generated using serial dilutions (0, 2.5, 5, 10 and 20 μg/ml) of human IgG probed with goat-anti human IgG-Cy5.

Internal calibration curve of the microarray was generated by probing a serial dilution of human IgG solution with goat-anti human IgG-Cy5 and the FI value of BSA probed with goat-anti human IgG-Cy5 was set as the first point of the internal calibration curve. Linear regression analysis revealed that all the calibration curves gave the good correlation coefficients (r^2^) ranging from 0.967 to 0.997 (Figure 2).

Eleven rSEPs on the microarray were probed with the patient sera. As a result (Table 2), all the rSEPs showed sensitivities of between 53% and 82% in recognizing acute-phase sera and of between 65% and 82% in recognizing convalescent-phase sera, and all the rSEPs except rRplA showed specificities of between 80% and 95%. Four rSEPs (rOmpA-2, rRplA, rRpsB, and rSdhB) showed sensitivities of ≥71% in recognizing both acute- and convalescent-phase sera. Also the summary of sensitivities of each protein to both acute- and convalescent-phase sera and specificity of each protein was calculated to generally evaluate its ability as a candidate antigen for diagnosis of FESF. As a result (Table 2), four rSEPs (rOmpA-2, rOmpB-3, rRpsB, and rSdhB) scored higher than the rest.

**Table 2:** The sensitivity and specificity of individual rSEPs on microarray probed with the acute-phase sera (n = 17) or convalescent-phase sera (n = 17)

	**Sensitivity (%)**		**Specificity**	Summary*
**Proteins**	**Acute-phase**	**Convalescent-phase**	**(%)**	**(%)**
rGroEL	65	65	80	209
rOmpA-2	71	82	85	238
rOmpB-3	65	82	90	237
rPrsA	53	76	95	224
rRh054_02285	71	65	90	225
rRplA	71	82	65	218
rRplY	65	82	85	232
rRpsB	71	71	95	236
rSdhB	82	76	90	249
rSurA	59	82	90	231
rYbgF	65	65	90	219

*The summary of sensitivities of each protein to both acute- and convalescent-phase sera and specificity of each protein was calculated to evaluate its ability as a candidate antigen for diagnosis of FESF.

### Relationship between specific IgG titers of sera and seroreactivity of rSEPs

**Figure 3:**
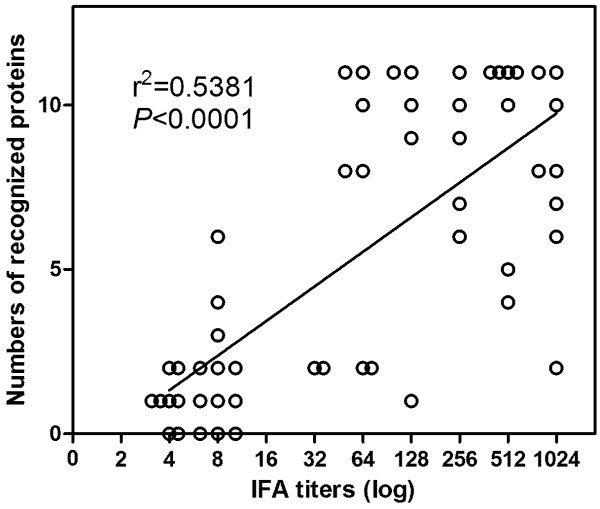
**Linear regression analysis to examine potential relationships between the IgG titers of FESF patient sera (n = 54) and the numbers of rSEPs recognized by these sera.** The IgG titer of each serum in IFA was log transformed. The analysis was conducted using GraphPad Prism 5 software (GraphPad Software, Inc., San Diego, CA).

Linear regression analysis revealed a significant positive correlation between the log-transformed IgG titers to *R. heilongjiangensis* in all the patient sera and the numbers of proteins recognized by these sera (Figure 3, r^2^ = 0.5381, *P* <0.0001, n = 54).

In addition, linear regression analysis revealed that significant correlations between the increase in the log-transformed IgG titers to *R. heilongjiangensis* for paired sera and the rising in RASIgG to rOmpA-2 (r^2^ = 0.2621, *P* = 0.0356) or rRpsB (r^2^ = 0.2838, *P* = 0.0277) in these sera (Table 3).

**Table 3:** **Linear regression analysis to examine potential relationships between the increased IgG titers to *R. heilongjiangensis *** **and the increased IgG level to individual rSEPs in paired sera**

**Protein name**	**Coefficients of correlations (r**^**2**^**)**	***P*** **value**
rGroEL	0.09251	0.2353
rOmpA-2*	0.2621	0.0356
rOmpB-3	0.01372	0.6544
rPrsA	0.1329	0.1502
rRh054_02285	0.002353	0.8533
rRplA	0.06482	0.3241
rRplY	0.003742	0.8156
rRpsB*	0.2838	0.0277
rSdhB	0.005894	0.7696
rSurA	0.05963	0.3449
rYbgF	0.04017	0.4405

*Statistically significant (*P* < 0.05) associations are marked.

## Discussion

In our previous study [9], eleven SEPs of *R. heilongjiangensis* were recognized as major seroreactive antigens and potential candidate antigens for serological diagnosis of FESF in microarray assay with *R. heilongjiangensis*-infected mouse sera. In the present study, their recombinant proteins fabricated on a microarray were assayed with paired sera from FESF patients so as to identify potential candidate antigens for serological diagnosis of FESF, as well as to explore the kinetic change of the specific antibodies to individual SEPs in FESF patients.

Firstly, these rSEPs were immunoblotted by paired sera from one FESF patient, and most of them showed a stronger reaction with the convalescent-phase serum than with the acute-phase serum, which suggested that more specific antibodies to these SEPs appeared in the convalescent-phase serum. This could not be quantitatively determined in the immunoblotting assay. However, the reactivity of each rSEP with individual sera was quantitatively determined by the microarray assay. In addition, the FI value of each protein probed with individual sera was interpolated with the calibration curve, which minimized variability in this quantitative determination so as to improve the within-slide and between-slide analytical precision.The individual rSEPs were analyzed by both immunoblot and microarray assay using the paired sera from patient 8. All of the rSEPs except OmpB-3 and YbgF, to which the RASIgG were higher in microarray, showed a stronger staining reaction in immunoblot assay (Figure 1B,C). The exception may be due to the different states of OmpB-3 and YbgF existing in different assays. OmpB-3 was stained very lightly in immonoblot while the RASIgG in the serum detected with microarray was big, which might be due to the tertiary structure of OmpB-3 on the microarray slide that might have exerted a steric effect to promote non-specific absorption of IgG from the sera, one effect that would not apply to the denatured OmpB-3 in the immunoblot assay. YbgF was stained strongly in immunoblot assay while the RASIgG in microarray assay was small. YbgF was denatured and might provide more epitopes to bind the specific antibodies in immonoblot assay, while it maintained its native structure and might provide less epitopes to bind the specific antibodies in the microarray assay.

In this microarray assay, only 5% to 20% of reference sera from the acute febrile patients without antibodies to *R. heilongjiangensis* reacted positively to individual rSEPs except rRplA, suggesting these rSEPs had a good specificity. The cross-reaction might be caused by the conservative SEPs such as the ribosomal protein RplA and patients from whom the reference sera were collected have suffered from other infection caused by bacteria which shared the conservative SEPs with *R. heilongjiangensis*. All of these rSEPs gave a sensitivity of over 65% to the convalescent-phase sera from FESF patients while five rSEPs (rOmpA-2, rOmpB-3, rRplA, rRplY, and rSurA) had a higher sensitivity of 82% to them. However, only rSdhB had a higher sensitivity of 82% to the acute-phase sera from FESF patients and the other rSEPs gave sensitivities of only between 53% and 71% to them.

The summary of sensitivity and specificity was calculated and four rSEPs (rOmpA-2, rOmpB, rRpsB, and rSdhB) had relatively higher scores. Also we found that the four rSEPs could recognize 63%, 63%, 74%, and 85% of the 19 FESF patient sera with lower IFA titer of ≤256 (2, 7, 5, and 5 of these sera have IFA titers of 32, 64, 128, and 256, respectively), respectively. When combination analysis of the data resulting from two, three, or four of the four rSEPs was performed (Table 4), and the patient was diagnosed as having FESF if the serum sample from him or her is positively recognized by at least one of the rSEPs, better sensitivities of between 76% and 94% to the acute-phase sera or between 82% and 100% to the convalescent-phase sera and acceptable specificities of between 75% and 90% were obtained. Our results suggest that the remarkable variation in immune recognition patterns for FESF require multi-antigen combination to cover the different antibody responses and thus achieve the highest possible test sensitivity. Serological tests are the easiest methods for the diagnosis of rickettsiosis but seroconversion is usually detected 7–15 days after disease onset [18]. Our combination assay could recognize as many as 94% of the acute-phase sera and hopefully diagnose FESF rapidly at the early stage of infection. Therefore, the four rSEPs may be considered as more likely candidate antigens for the serological diagnosis of FESF, especially rSdhB, with its sensitivity of 82% to the acute-phase sera and 76% to the convalescent-phase sera with specificity of 90%. Furthermore, refinement of the production of fusion molecules comprised of these SEPs and the reaction conditions of microarray assay described herein may lead to improve the sensitivity and specificity for the serodiagnosis of FESF. Epitopes in these proteins can be predicted using bioinformatics method and synthesized to evaluate their ability of diagnosis of FESF. Then the molecules fused with different combination of the selected epitopes may show better sensitivities and specificities.

**Table 4:** The sensitivity and specificity of the combination assays composed of different rSEPs in recognizing the acute-phase sera (n = 17) or convalescent-phase sera (n = 17)

	**Sensitivity (%)**		**Specificity**
**Proteins**	**Acute-phase**	**Convalescent-phase**	**(%)**
rOmpA-2&rOmpB-3	82	94	80
rOmpA-2&rRpsB	76	94	80
rOmpA-2&rSdhB	88	94	80
rOmpB-3&rRpsB	82	94	90
rOmpB-3&rSdhB	88	94	80
rRpsB&rSdhB	82	82	85
rOmpA-2, rOmpB-3&rRpsB	88	100	80
rOmpA-2, rOmpB-3&rSdhB	94	100	75
rOmpA-2, rRpsB&rSdhB	88	94	75
rOmpB-3, rRpsB&rSdhB	88	94	80
rOmpA-2, rOmpB-3, rRpsB&rSdhB	94	100	75

All of these rSEPs except rRh054_02285 and rSdhB probed with the convalescent-phase sera gave the same sensitivity or a higher sensitivity than probed with the acute-phase sera. This is not unexpected since the convalescent-phase sera had higher titers of antibodies to *R. heilongjiangensis*. We noticed that RASIgG to rRh054_02285 or rSdhB did decrease when probed with paired sera from some patients, indicating the specific IgG level to these proteins decreased quickly in some of these FESF patients during the convalescent phase.

Linear regression analysis revealed a significant positive correlation between the log-transformed specific IgG titers of FESF patient sera in IFA and the numbers of rSEPs recognized by these patient sera in microarray assay (Figure 3, *P* < 0.0001). This indicated that specific IgG levels to individual SEPs might contribute to the specific IgG titers to *R. heilongjiangensis* in FESF patient sera since they were major seroreactive antigens of *R. heilongjiangensis*. Moreover, the increase in IgG titers to *R. heilongjiangensis* for paired sera and the rising in RASIgG to rOmpA-2 or rRpsB in these sera were significantly correlated (Table 3, *P* < 0.05), indicating that antibodies to OmpA-2 or RpsB contributed more to the increase in IgG titers to *R. heilongjiangensis* than antibodies to other SEPs.

In the present study, the number of paired patient sera tested was small, which may influence the sensitivities and specificities of these rSEPs. Detection of specific IgM antibody to individual rSEPs might improve the sensitivity to acute-phase sera of FESF patients and unfortunately some paired patient sera were not enough to do this test. Therefore, it is necessary to get more serum samples of FESF patients for this microarray assay in the future.

## Conclusions

In conclusion, the eleven SEPs were serologically characterized with paired sera from FESF patients, and four rSEPs (rOmpA-2, rOmpB-3, rRpsB, and rSdhB) are more likely candidate antigens for the serological diagnosis of FESF. In addition, an optimized microarray composed with the four rSEPs may give an acceptable sensitivity for serological diagnosis of FESF during both the acute and convalescent phase.

## Competing interests

The authors declare that they have no conflict of interest.

## Authors’ contributions

YQ and WG carried out the experiments, data analyses and drafted the manuscript. XX assisted the analysis of data; JJ and YW provided the patient sera and helped to draft the manuscript; JJ and CD participated in its design and helped to draft the manuscript; BW designed the experiments and revised the manuscript. All authors read and approved the final manuscript.

## Pre-publication history

The pre-publication history for this paper can be accessed here:

http://www.biomedcentral.com/1471-2334/14/332/prepub
